# Beyond just correlation: causal machine learning for the microbiome, from prediction to health policy with econometric tools

**DOI:** 10.3389/fmicb.2025.1691503

**Published:** 2025-10-01

**Authors:** Issam Khelfaoui, Wenxin Wang, Hicham Meskher, Akram Ismael Shehata, Mohammed F. El Basuini, Mohamed F. Abouelenein, Houssem Eddine Degha, Mayada Alhoshy, Islam I. Teiba, Seedahmed S. Mahmoud

**Affiliations:** ^1^School of Public Health, Shantou University/Institute of Local Government Development, Shantou University, Shantou, China; ^2^Key Laboratory for Preparation and Application of Ordered Structural Materials of Guangdong Province, Department of Chemistry, Shantou University, Shantou, China; ^3^Institute of Marine Sciences, Shantou University, Shantou, China; ^4^Department of Animal and Fish Production, Faculty of Agriculture (Saba Basha), Alexandria University, Alexandria, Egypt; ^5^King Salman International University, El Tor, Egypt; ^6^Department of Insurance and Risk Management, College of Business, Imam Mohammad Ibn Saud Islamic University (IMSIU), Riyadh, Saudi Arabia; ^7^Department of Computer Science and Information Technologies, Faculty of New Technologies of Information and Communication, Kasdi Merbah Ouargla University, Ouargla, Algeria; ^8^Independent Researcher, Alexandria, Egypt; ^9^Department of Botany, Faculty of Agriculture, Tanta University, Tanta, Egypt; ^10^Department of Biomedical Engineering, College of Engineering, Shantou University, Shantou, China

**Keywords:** human microbiome, causal-ML, econometric methods, explainable artificial intelligence AI, policy translation

## Abstract

The human microbiome is increasingly recognized as a key mediator of health and disease, yet translating microbial associations into actionable interventions remains challenging. This review synthesizes advances in machine learning (ML) and causal inference applied to human microbiome research, emphasizing policy-relevant applications. Explainable ML approaches, have identified microbial drivers, guiding targeted strategies. Econometric tools, including instrumental variables, difference-in-differences, and panel data models, provide robust frameworks for validating causal relationships, while hybrid methods like Double Machine Learning (Double ML) and Deep Instrumental Variables (Deep IV) address high-dimensional and non-linear effects, enabling precise evaluation of microbiome-mediated interventions. Policy translation is further enhanced by federated learning, standardized analytical pipelines, and model visualization frameworks, which collectively improve reproducibility, scalability, and data privacy compliance. By integrating predictive power with causal rigor, microbiome research can move beyond observational associations to generate interventions that are biologically grounded, clinically actionable, and policy-ready. This roadmap provides a blueprint for translating mechanistic microbial insights into real-world health solutions, emphasizing interdisciplinary collaboration, standardized reporting, and evidence-based policymaking.

## 1 Introduction

Over the past two decades, our understanding of the human microbiome has undergone a profound transformation. Once considered a biological curiosity, the trillions of microorganisms inhabiting the human body are now recognized as active participants in health, disease, and therapeutic response. Microbiome research has evolved from descriptive catalogs of microbial diversity toward sophisticated analyses designed to predict, explain, and ultimately manipulate microbial dynamics for clinical and public health benefit. This evolution has been propelled by advances in high-throughput sequencing, computational biology, and, increasingly, machine learning (ML). However, despite the power of ML to detect complex microbiome–health associations, a crucial limitation remains: correlation does not imply causation. Without rigorous causal inference, predictive models may fail to generalize, interventions may miss their intended targets, and policy decisions may rest on uncertain foundations. This limitation is not only of academic concern, industry and policy stakeholders have already recognized the strategic value of translating microbiome insights into actionable programs.

**Figure 1:**
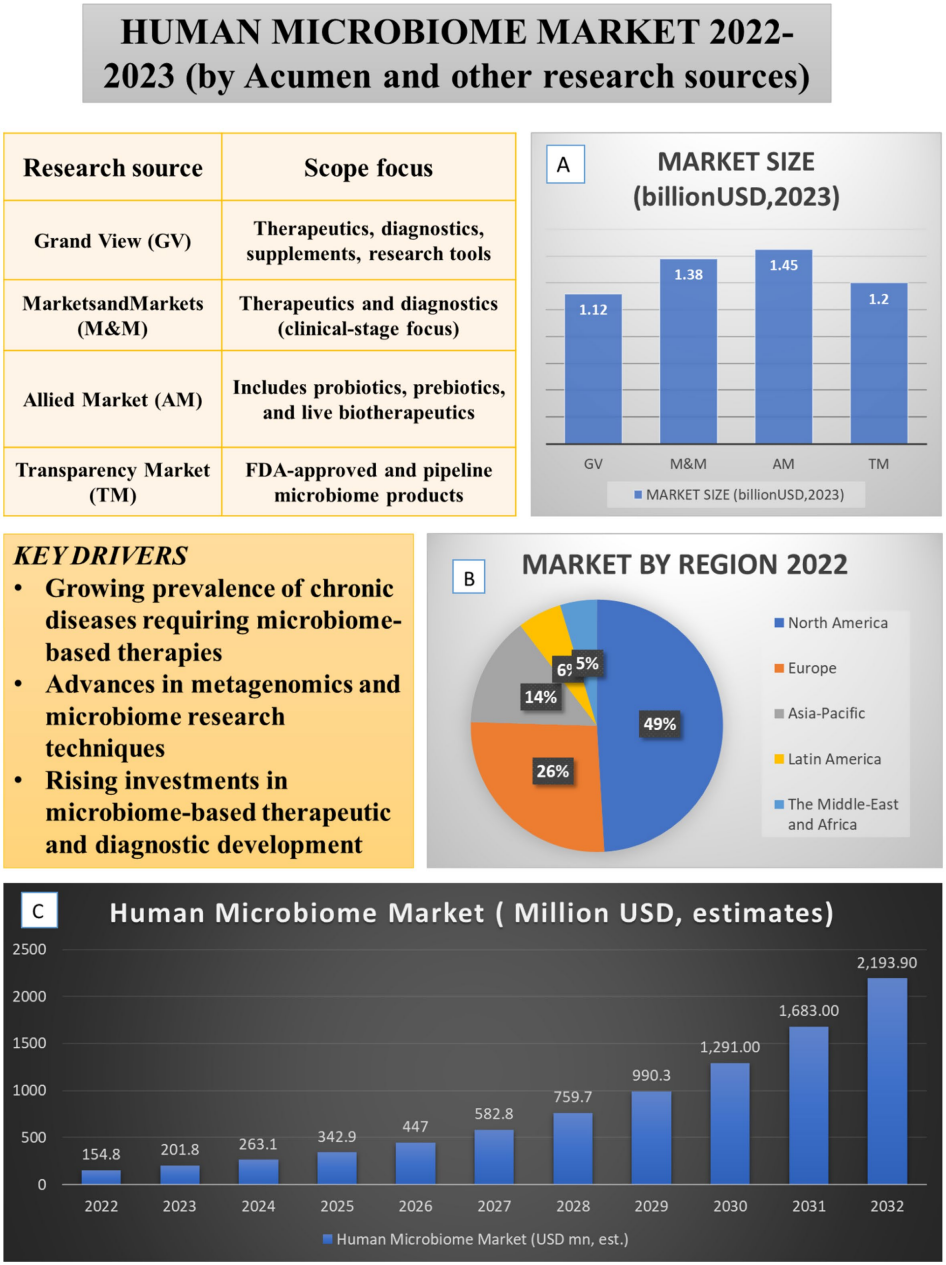
Global human microbiome market landscape (2022–2023): size, regional distribution, and key drivers. The figure integrates key drivers, market size **(A)**, regional distribution **(B)**, and projected growth of the human microbiome market **(C)**. Market drivers include the rising prevalence of chronic diseases requiring microbiome-based therapies, advances in metagenomics and causal ML techniques, and increased investments in therapeutic and diagnostic development. Comparative analyses from multiple research sources (GV, M&M, AM, TM, 2025 reports) estimate the 2023 market size between USD 1.12–1.45 billion. Data from Acumen Research and Consulting show that in 2022, North America (49%) led the regional market, followed by Europe (26%) and Asia-Pacific (14%), with projected global expansion from USD 154.8 million in 2022 to USD 2193.9 million by 2032.

This composite Figure 1 summarizes the current state and projected growth of the human microbiome market based on multiple research sources. Panel A shows the estimated market size in 2023 across four major firms, highlighting differences in scope and segmentation. Panel B presents the regional market share in 2022, with North America dominating due to regulatory leadership and investment. Panel C illustrates projected market growth from 2022 to 2032, based on Acumen’s forecast. Key drivers of market expansion include the rising prevalence of chronic diseases requiring microbiome-based therapies, advances in metagenomics, and increasing investments in therapeutic development.

### 1.1 Microbiome and health

The human microbiome has emerged as a critical determinant of health and disease. Foundational studies have demonstrated its involvement in conditions ranging from colorectal adenoma (Goedert et al., 2015) to cirrhosis progression (Bajaj et al., 2020). Advances in high-throughput sequencing and ML have further revealed predictive potential across diverse contexts, including stratifying rheumatoid arthritis treatment responses (Gupta et al., 2021), identifying high-risk myeloma signatures (Feinman et al., 2023), and detecting gastric cancer trends (Zhang et al., 2025). Interpretable ML models have also been successfully applied to classify diabetes subtypes (Gou et al., 2020) and identify obesity-associated microbial markers (Zeng et al., 2019).

Despite these advances, predictive performance does not always translate into actionable insight. For example, short-chain fatty acids (SCFAs) in fecal samples often fail to reliably predict tumor development (Sze et al., 2019). Viral sequence analyses remain challenging due to gaps in reference databases and assembly biases (Kieft et al., 2020; Ren et al., 2017). Integrative studies combining ocular microbiome and metabolomic data further highlight the complexity of cross-domain microbiome research (Gao et al., 2025).

### 1.2 The causality gap

Correlational microbiome studies remain vulnerable to confounding and bias. For instance, tuberculosis medications can distort predictions of inflammatory states (Wipperman et al., 2021), batch effects in oral microbiome datasets introduce noise (Rupf et al., 2018), and sputum extraction methods can bias microbial profiles (Oriano et al., 2019). In non-alcoholic fatty liver disease (NAFLD), metabolome–microbiome associations often lack clear directionality (Schwenger et al., 2024), while antimicrobial use can artificially skew microbial ratios (Hao et al., 2015). Similarly, obesity-associated cytokines can obscure links to osteoarthritis (Kurz et al., 2025), and dysbiosis in polycystic ovary syndrome (PCOS) independent of Body Mass Index BMI underscores the heterogeneity of microbiome–disease interactions (Mammadova et al., 2020).

Machine learning–enhanced causal frameworks are increasingly being applied to address these limitations more effectively than traditional statistical methods. Double Machine Learning (Double ML) has been employed to control for high-dimensional confounders in microbiome disease associations (Chen et al., 2024), while causal forests have been used to quantify heterogeneous treatment effects in nutritional studies (Ben-Yacov et al., 2023). Nevertheless, challenges remain; for example, microbiome-based predictions for Long COVID have struggled to achieve robust performance (Calvani et al., 2024), highlighting the need for more sophisticated ML-driven mediation analyses and integration of domain knowledge. These observations emphasize that predictive accuracy alone is insufficient when the ultimate goal is intervention or policy change. Without establishing causality, strategies may be ineffective or even harmful.

### 1.3 Contributions of this review

This review aims to bridge the gap between predictive ML and actionable causal insights in microbiome research. We systematically map the integration of advanced causal inference techniques with econometric tools, focusing on methods such as Double ML for evaluating microbiome-mediated treatment effects (Wu et al., 2022) and high-dimensional mediation analysis for exploring microbial community dynamics (Chen et al., 2024). Econometric frameworks, including directed acyclic graphs (DAGs) for causal mapping in Alzheimer’s disease microbiome interactions (Qiu et al., 2024) and deep restricted Boltzmann machines (RBMs) for microbial network inference (Sokolovska et al., 2020), are highlighted for their potential to formalize causal assumptions and reduce bias.

We further examine emerging computational platforms, such as Microbiome Causal Machine Learning MiCML, that operationalize causal ML for clinical decision-making (Koh et al., 2025). Translational applications are reviewed, including hyperuricemia diagnostic flowcharts (Miyajima et al., 2024), model cards for hepatitis B virus (HBV)-related hepatocellular carcinoma (Hu B. et al., 2024), and malnutrition intervention frameworks (Portlock et al., 2025). These approaches are then linked to policy-relevant contexts, such as cardiovascular disease risk prediction (Warmbrunn et al., 2024), COVID-19 microbiome-informed guidelines (Bucci et al., 2023), and immunotoxicity trial design (Liu et al., 2024).

The human microbiome plays a critical role in health and disease, yet translating complex microbial data into actionable interventions remains challenging. Current studies often rely on correlational analyses or single-method approaches, limiting causal understanding and policy applicability. To address this gap, we present the first systematic review integrating causal machine learning approaches with econometric methodologies in microbiome science. We aim to provide a rigorous, policy-relevant framework that translates microbiome discoveries into robust, intervention-ready evidence for researchers, clinicians, data scientists, and policymakers seeking targeted, equitable, and evidence-based applications.

## 2 Materials and methods

This systematic review was conducted following the Preferred Reporting Items for Systematic Reviews and Meta-Analyses (PRISMA) 2020 guidelines (Page et al., 2021), with the objective of examining how causal inference methods and machine learning techniques have been jointly applied in human microbiome research with clinical or policy relevance. The search strategy targeted peer-reviewed studies published between January 2015 and May 2025, reflecting the recent surge in computational approaches capable of integrating high-dimensional microbiome data with rigorous causal identification frameworks (Ruggiero et al., 2021; Sohrabi et al., 2021).

Two electronic databases were searched: PubMed and Dimensions.ai; the choice of both databases is intensively proven in research, and they are widely used (Falagas et al., 2008; Lee et al., 2023; Mouratidis, 2019; Ossom Williamson and Minter, 2019; Pan et al., 2018). Boolean queries were constructed to capture literature at the intersection of microbiome science, causal inference, and machine learning. The microbiome component included terms such as “microbiome,” “gut microbiota,” “gastrointestinal microbiome,” and “16S rRNA.” Causal inference terms encompassed “instrumental variable,” “Mendelian randomization,” “difference-in-differences,” and “causal machine learning,” while the machine learning component included “machine learning,” “random forest,” “deep learning,” “double ML,” “causal forest,” and “Bayesian additive regression trees.” To exclude animal-only or *in vitro* studies, the filter NOT “animal” [*tiab*] was applied. The final search strings were adapted to the syntax and indexing systems of each database.

The initial search identified 571 records, including 70 from PubMed and 501 from Dimensions.ai. All records were exported in MEDLINE (.nbib) format to preserve structured metadata, including MeSH terms, author affiliations, and structured abstracts. Duplicates were removed using Rayyan.ai’s automated detection tool, followed by manual verification of ambiguous matches, resulting in 477 unique records for title and abstract screening.

Screening was performed in two stages. First, titles and abstracts were reviewed against four inclusion criteria: studies must (1) focus on the human microbiome, (2) employ a causal inference method, (3) incorporate at least one machine learning approach, and (4) address clinical or public health outcomes. This process yielded 73 records for full-text assessment. In the second stage, full texts were reviewed in detail to verify eligibility. Studies were excluded if they applied purely causal inference methods without any machine learning component (*n* = 46), focused exclusively on animal or *in vitro* models, used predictive models without causal framing, or were limited to exploratory or correlational analyses. Additional exclusion criteria included non-English language publications and review articles.

A total of 19 studies met all inclusion criteria. These comprised original research articles explicitly combining causal inference and machine learning within human microbiome contexts, reporting findings relevant to health outcomes. Among these, 15 studies demonstrated particularly strong policy relevance, characterized by discussion of translational applications, public health interventions, or clinical decision-making pathways. These were selected for deeper thematic analysis in the results and discussion sections.

For each included study, a standardized data extraction protocol was applied. Extracted variables included bibliographic information, study design, sample size and population characteristics, type of microbiome data (e.g., 16S rRNA sequencing, metagenomics), causal inference method used, type of machine learning approach, specific health or policy outcomes studied, and statistical validation strategies such as sensitivity analyses or falsification testing. Where applicable, we documented whether studies addressed potential sources of bias, implemented robustness checks, or discussed limitations in causal interpretation. Special attention was given to identifying whether authors proposed pathways for translating findings into actionable clinical or policy recommendations.

**Figure 2:**
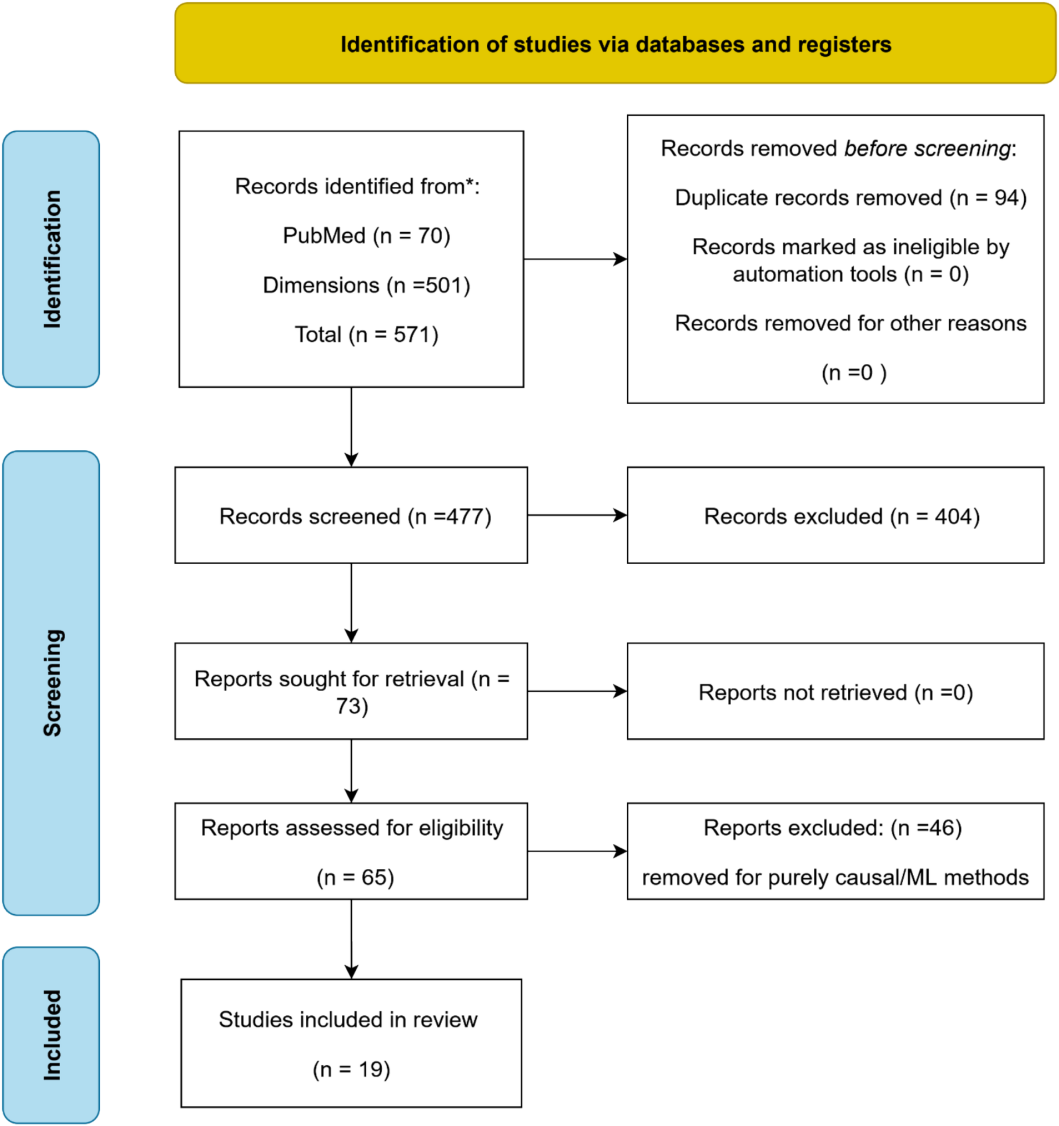
PRISMA diagram for the systematic review research methodology.

All steps in the review process were documented to ensure reproducibility. Screening and full-text review were managed entirely within Rayyan.ai, which preserved all metadata in MEDLINE format. The overall selection process is summarized in a PRISMA 2020-compliant flow diagram (Figure 2), illustrating the numbers of records identified, screened, assessed for eligibility, and included, as well as reasons for exclusion at each stage. This transparent approach ensured methodological rigor and facilitated reproducibility.

## 3 Machine learning for microbiome prediction

### 3.1 Supervised learning in microbiome studies

Supervised machine learning approaches have become indispensable tools for translating complex microbiome data into clinically actionable insights (Cammarota et al., 2020; Malakar et al., 2024). The field has progressed from early correlation studies to sophisticated predictive models that account for population heterogeneity. For example, Neri-Rosario et al. (2023) developed ethnicity-specific models for type 2 diabetes prediction in Mexican cohorts, addressing critical limitations in generalizability that had affected earlier models. These advances built upon foundational work by Gou et al. (2020), who established interpretable frameworks for microbial biomarker discovery in diabetes. Among supervised algorithms, Random Forest classifiers have demonstrated particular utility in microbiome applications due to their ability to handle high-dimensional taxonomic data. Goedert et al. (2015) applied Random Forests to detect colorectal adenoma, achieving robust classification despite sparse data, while Zeng et al. (2019) used the same approach for obesity subtyping through microbial signatures. However, these models may face challenges with population transferability, as highlighted by Koduru et al. (2022) who observed performance degradation when models trained on North American cohorts were applied to South Asian populations.

Gradient boosting methods, including eXtreme Gradient Boosting (XGBoost), have emerged as powerful alternatives, particularly for their superior regularization capabilities. Gou et al. (2020) leveraged these advantages to develop a type 2 diabetes prediction model that outperformed logistic regression by 23% in precision. Model interpretability has been further enhanced through techniques such as SHapley Additive exPlanations (SHAP) analysis, exemplified by Wu et al. (2022), who investigated berberine’s cholesterol-modulating effects through microbial mediators. Deep learning approaches are now pushing the boundaries, with transformer models decoding complex gut-brain axis interactions in Alzheimer’s disease (Qiu et al., 2024) and platforms like MiCML making these tools more accessible for translational research (Koh et al., 2025).

**Figure 3:**
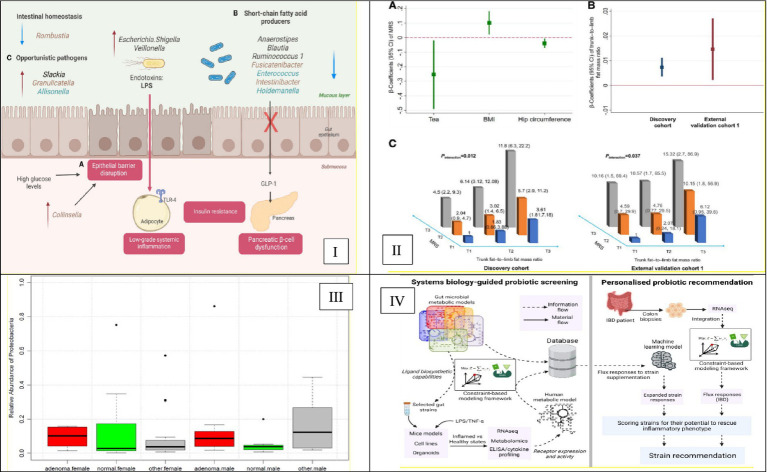
Advancements in supervised machine learning for microbiome-based disease prediction. **(I)** Illustrates ethnicity-specific gut microbiome signatures linked to type 2 diabetes risk. It highlights the necessity of incorporating ethnic diversity into predictive models to improve their generalizability and accuracy. This is Figure 3 from Neri-Rosario et al. (2023). **(II)** Presents an interpretable microbial risk score (MRS) that identifies gut microbiome features predictive of type 2 diabetes, demonstrating the potential of ML models to reveal actionable biomarkers. This is Figure 2 from Gou et al. (2020). **(III)** Depicts a Random Forest classifier that analyzes gut microbiome data to detect colorectal adenoma, illustrating the model’s utility for microbiome-based disease classification. This is Figure 4 from Goedert et al. (2015). **(IV)** Highlights the performance degradation of microbiome-based models when applied to South Asian populations, addressing the critical challenge of ensuring model generalizability across diverse groups. This is Figure 3 from Koduru et al. (2022).

This composite Figure 3 collectively underscores the evolution and challenges of applying supervised machine learning techniques to microbiome-based disease prediction. Panel A emphasizes the importance of ethnicity-specific models to enhance prediction accuracy in diverse populations. Panel B showcases the development of interpretable models that identify actionable microbial biomarkers. Panel C demonstrates the successful application of Random Forest classifiers in disease classification tasks. Finally, Panel D highlights the challenges of transferring models across different populations, stressing the need for inclusive and adaptable models in microbiome research. The main supervised learning techniques and their applications in microbiome research are summarized in Table 1, which provides a reference for classification, feature selection, survival analysis, and interpretability applications. This table highlights method-specific best practices, example use cases, and corresponding references for reproducibility.

**Table 1:** Supervised learning techniques in microbiome research.

Technique	Best for (technique goal and microbiome research goal)	Example use case	References
Random Forest (RF)	Classification tasks with high-dimensional microbiome data; identifying microbial biomarkers	Type 2 diabetes (T2D) classification	Neri-Rosario et al. (2023)
		Colorectal adenoma detection	Sze et al. (2019)
		Obesity-associated dysbiosis	Mammadova et al. (2020)
		Polycystic ovary syndrome (PCOS) microbiota characterization	Oriano et al. (2019)
		Kidney transplant outcome prediction	Noda et al. (2024)
		Malnutrition-cognition links via microbiota	Portlock et al. (2025)
		Personalized diet effects on cardiometabolic markers	Wu et al. (2022)
		Depression-linked gut dysbiosis	Chen et al. (2021)
		Gut microbes predicting rheumatoid arthritis (RA) treatment response	Hu M. et al. (2024)
		Autism spectrum disorder (ASD) subtypes linked to gut microbiome	Schwenger et al. (2024)
		Long COVID prediction	Hu B. et al. (2024)
		Endometrial cancer prediction from gut microbiota	Chang et al. (2024)
Least Absolute Shrinkage and Selection Operator (LASSO) Regression	Feature selection for high-dimensional data; identifying sparse microbial signatures	Stroke recovery biomarker selection	Dang et al. (2021)
		Non-alcoholic fatty liver disease (NAFLD)-linked microbial biomarkers	Yaskolka Meir et al. (2023)
		Immunotherapy toxicity-microbiome associations	Zeng et al. (2022)
Support Vector Machine SVM	Binary/multiclass classification; robust to noise in microbiome data	Sputum DNA extraction optimization	Kim et al. (2020)
		Predictive models for inflammatory bowel disease (IBD) severity	Ben-Yacov et al. (2023)
		Crohn’s disease biomarker selection Support Vector Machine-Recursive Feature Elimination (SVM-RFE)	Qu et al. (2024)
XGBoost	Handling imbalanced datasets; high-performance gradient boosting	T2D subtype classification	Gupta et al. (2021)
		Gastric cancer risk prediction (XGBoost and LASSO)	Zhang et al. (2025)
		Gut microbiome predicts immune checkpoint inhibitor toxicity	Jung et al. (2024)
		Acute pancreatitis-microbiome risk modeling	Liu et al. (2024)
Deep Neural Networks (DNN)	Modeling complex nonlinear relationships in multi-omics data	Neurodegenerative disorder prediction	Neri-Rosario et al. (2023)
Logistic Regression	Interpretable models for binary outcomes (e.g., disease risk)	Stroke risk prediction	Ma et al. (2025)
Elastic Net	Combines LASSO and ridge regression for correlated features	Polyphenol effects on DNA methylation age	Comba et al. (2024)
SHAP and RF	Interpretable ML for feature importance in microbiome studies	Berberine’s microbiome-mediated cholesterol effects	Wu et al. (2022)
Cox Regression	Survival analysis with microbiome covariates	Microbiome mediation in HBV-hepatocellular carcinoma (HCC) progression	Yang et al. (2025)

### 3.2 Unsupervised learning approaches and their challenges

Unsupervised learning methods are widely used to explore microbial community structure without prior assumptions, enabling the discovery of patterns in high-dimensional microbiome data. However, their application is complicated by technical artifacts, methodological choices, and the inherent complexity of microbial ecosystems (Busato et al., 2023; Cai et al., 2017; Dutta et al., 2022; Hao et al., 2011). Technical biases can dominate clustering outcomes, leading to misleading biological interpretations. Schwab et al. (2019) showed that sample storage conditions significantly influenced clustering patterns in breast milk microbiota, with differences in freeze–thaw cycles and preservation time outweighing biological variation. Such pre-analytical factors underscore the need for standardized protocols across studies to ensure reproducibility. The utility of discrete microbial groupings, such as enterotypes, has also been questioned. Zeng et al. (2019) demonstrated that obesity-related gut microbiome changes often follow continuous gradients rather than distinct clusters, suggesting that apparent enterotypes may arise from algorithmic artifacts rather than true ecological boundaries. This highlights the risk of imposing categorical structure on inherently continuous data.

In clinical contexts, analytical decisions further shape results. Schwenger et al. (2024) found that distance metric choice, Bray–Curtis versus UniFrac, led to divergent microbial subtypes in non-alcoholic fatty liver disease (NAFLD), altering clinical interpretations. Additionally, variability in DNA extraction methods has been shown to affect microbial profiles; Oriano et al. (2019) reported that lysis efficiency differences across protocols skewed abundance estimates, particularly for Gram-positive bacteria, reinforcing the need for methodological transparency.

Dimensionality reduction techniques remain valuable for visualizing community structure. Principal component analysis (PCA) and principal coordinate analysis (PCoA) help identify major sources of variation, while t-distributed stochastic neighbor embedding (t-SNE) has been used to resolve fine-scale dynamics, such as microbial shifts during tuberculosis treatment (Wipperman et al., 2021). However, nonlinear methods require careful parameterization to avoid overinterpretation of local structures. Integrating microbiome data with host multi-omics profiles enhances biological insight. In multiple sclerosis research, combining microbial composition with metabolomic data revealed associations between taxa like *Akkermansia* and immunomodulatory metabolites, suggesting functional host–microbe interactions (Sheng et al., 2024). Co-occurrence networks, such as those generated by Microbial Co-occurrence Network Analysis (MiCA), further enable the identification of keystone taxa linked to environmental exposures (Koduru et al., 2022), though inferred correlations must be interpreted cautiously due to potential confounding.

Emerging generative models, including Generative Adversarial Networks (GANs), are beginning to simulate microbiome-immune dynamics *in silico*, offering new avenues for hypothesis generation and data augmentation. While still in early development, these approaches reflect a growing shift toward more sophisticated, integrative frameworks. In sum, unsupervised methods provide essential tools for exploratory microbiome analysis, but their results are highly sensitive to technical and analytical choices. Rigorous standardization, transparent reporting, and cautious interpretation are critical to ensure biological validity (Table 2).

**Table 2:** Unsupervised learning techniques in microbiome research.

Technique	Best for (technique goal and microbiome research goal)	Example use case	References
Principal component analysis (PCA)	Dimensionality reduction; identifying dominant microbial variation patterns	Breast milk microbiota clustering	Schwab et al. (2019)
		Immune thrombocytopenia microbiota clustering	Li et al. (2024)
		High-dimensional confounding in mediation analysis (PCA and network analysis)	Gao et al. (2025)
PCA and k-Means	Clustering microbiome data into distinct groups	Oral microbiome clustering	Rupf et al. (2018)
		Thromboangiitis obliterans microbiota clustering	Mao et al. (2023)
Latent Dirichlet Allocation (LDA)	Probabilistic topic modeling for microbiome “enterotypes”	Osteoarthritis enterotype classification	Kurz et al. (2025)
k-Means Clustering	Partitioning microbiome samples into discrete subgroups	Prostatitis microbiome enterotyping	Galiwango et al. (2022)
		Inflammatory dermatoses enterotyping	Midya et al. (2023)
PCoA	Beta-diversity visualization; sample dissimilarity mapping	Penile microbiota in HIV + men	Chen et al. (2025)
		Thromboangiitis obliterans microbiota clustering	Mao et al. (2023)
t-SNE	Nonlinear visualization of high-dimensional microbiome data	Trimethylamine N-oxide (TMAO)-diabetic retinopathy network clustering	Baranzini (2025)
Multi-omics Integration	Combining microbiome data with other omics	Multiple sclerosis (MS) microbiome-host interactions	Sheng et al. (2024)
Co-occurrence Networks (MiCA)	Microbial interaction networks; identifying keystone taxa	Lead exposure and childhood microbiome clustering	Koduru et al. (2022)
Deep Learning (GANs)	Synthetic data generation for microbiome-immune simulations	Simulated probiotic-immune interactions	Koduru et al. (2022)

### 3.3 Panel models for longitudinal microbiome analysis

Longitudinal study designs are essential for capturing the dynamic nature of host-microbiome interactions, enabling researchers to track microbial changes within individuals over time and link them to clinical outcomes. In this context, panel data models, commonly used in econometrics, have emerged as powerful tools for analyzing repeated microbiome measurements, allowing for the separation of within-subject temporal changes from between-subject variability. Bucci et al. (2023) applied such models to serial gut microbiome data from COVID-19 patients, revealing that significant dysbiosis, marked by loss of commensal taxa and reduced diversity, often preceded the onset of severe clinical deterioration. This temporal precedence suggests that microbiome destabilization may not merely reflect disease severity but could contribute to adverse outcomes, highlighting the potential of longitudinal modeling to uncover predictive microbial signatures.

A key advantage of panel models lies in their ability to control for unmeasured, time-invariant confounders, such as host genetics or early-life microbial colonization by treating each individual as their own control (Hill et al., 2020; Murayama and Gfrörer, 2024; Pesaran and Zhou, 2018; Streeter et al., 2017). Fixed-effects models eliminate these confounders through within-subject centering, making them ideal for detecting transient microbial shifts associated with interventions or disease flares. In contrast, random-effects models assume individual differences are random and can be modeled as part of the variance structure, offering greater efficiency when assessing population-level trends. Bogart et al. (2019) demonstrated how both approaches can be applied to microbiome data, showing that fixed-effects frameworks improve causal interpretability by isolating persistent microbial signals from noise, particularly in chronic conditions where baseline differences between individuals are substantial.

These models gain even greater power when combined with econometric techniques such as lagged variables, difference-in-differences, or instrumental variables, which help strengthen causal inference in observational settings. For example, incorporating lagged microbial states allows researchers to assess whether prior community composition influences future health outcomes a critical step in moving beyond correlation toward mechanistic understanding. Moreover, panel approaches can be adapted to handle the compositional nature of microbiome data through log-ratio transformations and integrated with regularization methods to manage high dimensionality. Despite their strengths, panel models require dense and consistent sampling to reliably capture temporal dynamics, and their assumptions may be challenged by nonlinear microbial trajectories or missing data. Nevertheless, their increasing use reflects a broader shift toward rigorous, theory-informed analysis in microbiome research. By leveraging longitudinal structure and controlling for confounding at the individual level, panel models offer a robust framework for uncovering the temporal logic of microbiome-host interactions in health and disease. These approaches, together with joint longitudinal-survival models and mixed-effects analyses, are detailed in Table 3, which outlines panel model techniques, their best-use scenarios, example studies, and associated machine learning approaches.

**Table 3:** Panel models for longitudinal microbiome analysis.

Technique	Best for	Example use case	References	Machine learning method
Joint Modeling	Linking longitudinal biomarker trajectories with time-to-event outcomes	Predicting COVID-19 severity based on gut microbiota trajectories	Bucci et al. (2023)	Joint longitudinal-survival modeling
Negative Binomial Mixed-Effects	Modeling count data with overdispersion and repeated measures	Predicting *C. difficile* recurrence using longitudinal microbiome data	Dawkins et al. (2022) and Yirga et al. (2020)	Generalized linear mixed effects
Multimodal Mediation	Analyzing time-structured indirect effects	Decoupling microbiome-immune-temporal dynamics in chronic diseases	Jiang et al. (2025)	Structural equation modeling (SEM)
MiCML Platform	Integrating multi-omics time-series data to quantify treatment effects	Antibiotic-induced microbiome shifts and host response in tuberculosis (TB) therapy	Koh et al. (2025)	Bayesian hierarchical modeling
Cox Proportional Hazards (PH) Model	Survival analysis with time-varying microbiome covariates	Vaginal microbiome and spontaneous preterm birth risk	Chen et al. (2022)	Proportional hazards regression
Mixed-Effects Models	Modeling repeated measures with subject-specific random effects	Penile microbiome dynamics and HPV persistence	Onywera et al. (2020)	Linear/logistic mixed effects
Structural Equation Modeling (SEM) (Path Analysis)	Testing hypothesized causal pathways in longitudinal data	Polyphenol-microbiome-aging links (DNA methylation clocks)	Yaskolka Meir et al. (2023)	Latent variable modeling

### 3.4 Hybrid machine learning methods

Hybrid machine learning approaches, those that strategically combine supervised and unsupervised techniques or integrate causal inference frameworks, are gaining traction in microbiome research as powerful tools to bridge the gap between pattern discovery and biological interpretability. By leveraging the exploratory strengths of unsupervised learning with the predictive precision of supervised models, these methods enable robust feature extraction from high-dimensional microbiome data while maintaining the ability to link microbial signatures to clinically relevant outcomes. This dual capacity is particularly valuable in complex host-microbe systems where both unknown community structures and defined phenotypic endpoints coexist. For example, Wipperman et al. (2021) employed a hybrid strategy combining t-SNE for dimensionality reduction with Random Forest classification to analyze longitudinal gut microbiome data from tuberculosis patients. This approach not only revealed dynamic microbial community trajectories during antimicrobial treatment but also successfully classified patients into distinct inflammatory response clusters, demonstrating how visualization and prediction can be synergistically combined to yield both mechanistic insight and clinical utility.

Beyond classification, hybrid models are increasingly used to uncover latent biological subtypes and assess their association with disease progression. One such method integrates latent Dirichlet allocation (LDA), a topic modeling technique originally developed for text analysis, with regression frameworks to identify microbiome-derived “topics” or co-abundance modules and test their association with clinical variables. This approach has been applied to osteoarthritis research, where LDA was used to define microbiome subtypes based on taxonomic co-occurrence patterns, which were then linked to pain severity, joint function, and systemic inflammation through multivariate regression (Kurz et al., 2025). By treating microbial communities as mixtures of underlying ecological themes, akin to topics in a document, this method captures nuanced, overlapping community states that traditional clustering might overlook. The integration with regression further allows for statistical inference, enabling researchers to quantify the contribution of each microbial topic to phenotypic variation while adjusting for confounders such as age, diet, and medication use.

More recently, hybrid frameworks incorporating causal inference have emerged to address the fundamental challenge of distinguishing correlation from causation in microbiome studies. Mendelian randomization (MR), which uses genetic variants as instrumental variables to infer causal relationships, has been paired with principal component analysis (PCA) to explore bidirectional interactions between host genetics and the gut microbiome. In immune thrombocytopenia (ITP), this MR-PCA approach revealed that host genetic variation influences microbial composition, particularly within the *Lachnospiraceae* and *Ruminococcaceae* families, while also suggesting feedback effects whereby specific microbial profiles modulate immune gene expression and platelet regulation (Li et al., 2024). Such integrative designs move beyond associative modeling to provide evidence for directional, potentially causal pathways, offering a more robust foundation for therapeutic targeting. As microbiome research shifts from descriptive analyses to mechanistic and interventional inquiry, hybrid machine learning methods will play an increasingly central role in transforming complex data into actionable biological knowledge. These hybrid approaches, their best-use scenarios, example applications, and corresponding machine learning methods are summarized in Table 4. They exemplify the capacity of modern computational pipelines to integrate predictive modeling with causal validation, offering actionable insights for precision medicine and policy-informed interventions.

**Table 4:** Hybrid machine learning techniques in microbiome research.

Technique	Best for (technique goal and microbiome research goal)	Example use case	References	ML method
LDA and Regression	Topic modeling for microbial sub-communities and predicting host phenotypes	Osteoarthritis enterotypes linked to clinical outcomes	Kurz et al. (2025)	Hybrid (LDA and Generalized Linear Models GLM)
t-SNE and Random Forest	Visualizing high-dimensional microbiome data and classifying disease states	TB treatment trajectories stratified by inflammatory markers	Lu et al. (2023)	Hybrid (t-SNE and RF)
MR and PCA	Causal inference and dimensionality reduction for bidirectional host-microbiome links	Immune thrombocytopenia and gut microbiome interactions	Li et al. (2024)	Hybrid (MR and PCA)
Random Forest and PCoA	Classification and beta-diversity visualization for longitudinal dynamics	HIV-associated penile microbiome clustering	Galiwango et al. (2022)	Hybrid (RF and PCoA)
Deep Restricted Boltzmann Machine (Deep RBM) and Causal Inference	Modeling nonlinear microbiome-host interactions with causal validation	Bivariate microbiome-metabolite networks in metabolic disorders	Qiu et al. (2024)	Hybrid (Deep Learning and Causal)
DNN and Interpretability	High-dimensional feature extraction and mechanistic insights	G-protein-coupled receptors (GPCRs) -microbiome links in Alzheimer’s disease	Qiu et al. (2024)	Hybrid (DNN and SHAP)
XGBoost and MR	Predictive modeling and genetic causal validation	Hyperuricemia prediction with microbiome features	Miyajima et al. (2024)	Hybrid (XGBoost and MR)
Network Analysis and Random Forest	Microbial co-occurrence networks and phenotype classification	Multi-ethnic cardiovascular disease (CVD)-microbiome associations	Warmbrunn et al. (2024)	Hybrid (Network and RF)
VirFinder and Random Forest	Viral sequence detection and host-disease prediction	Viral contig identification in metagenomic data	Ren et al. (2017)	Hybrid (k-mer and RF)
VIBRANT and MR	Viral functional annotation and causal pathway validation	Viral functions in liver cirrhosis progression	Kieft et al. (2020)	Hybrid (k-mer and MR)
Joint Modeling and MR	Longitudinal data integration and causal inference	Gut microbiota trajectories in COVID-19 severity	Chen et al. (2024)	Hybrid (Joint Model ad MR)

## 4 Causal inference with econometrics in microbiome research

This section examines the methodological landscape of causal ML in microbiome science, outlining how approaches such as double ML, instrumental variables, and federated designs are being adapted to address high-dimensional and heterogeneous data. We highlight how these innovations provide new strategies to overcome persistent challenges in causal inference.

### 4.1 Instrumental variables: uncovering causal pathways

Instrumental variable (IV) methods have been crucial for causal inference in econometrics, but microbiome research poses unique challenges due to high dimensionality and compositional data structures. Recent machine learning–enhanced approaches, including LASSO-IV (Chen et al., 2025) and causal forests (Miyajima et al., 2024), enable the identification of valid instruments in complex microbial datasets, allowing researchers to isolate causal effects with higher precision. Such methods have proven particularly effective in pharmacomicrobiomics studies, exemplified by Wu et al. (2022), who demonstrated berberine’s cholesterol-lowering effects mediated through specific microbial pathways.

Microbiome-specific IV adaptations, including compositional Mendelian randomization (Zeng et al., 2019) and phylogenetic directed acyclic graphs (Qiu et al., 2024), further strengthen causal inference by considering taxonomic and evolutionary dependencies. In addition, viral sequence data have been leveraged as instruments to control for bacteriophage-mediated confounding, opening novel opportunities to investigate host-microbe-virus interactions (Kieft et al., 2020). As summarized in Table 5, these innovations represent the key methodological advancements enabling robust causal inference in microbiome studies.

**Table 5:** Key innovations and methodological advancements in causal inference for microbiome studies.

Method	Breakthrough	Application	References
SHAP	Quantifies taxon-level contributions	Diabetes/HCC risk prediction	Hu B. et al. (2024) and Wu et al. (2022)
Biological DAGs	Incorporates host–microbe interactions	Neurodegenerative/IBD diseases	Katsidzira and Misselwitz (2025) and Qiu et al. (2024)
Adversarial Validation	Tests robustness to unobserved confounders	Infectious disease/toxicology	Wipperman et al. (2021)
Double ML	Debiases treatment effects in high-dimensional data	Personalized medicine/nutrition trials	Ben-Yacov et al. (2023) and Wu et al. (2022)
DeepIV	Neural networks for nonlinear IV estimation	Microbiome causal inference, GPCR-microbiome networks, viral-bacterial interactions	Bucci et al. (2023), Koh et al. (2025), and Qiu et al. (2024)
Meta-Learners	Estimates heterogeneous treatment effects	Precision medicine in rheumatology	Gupta et al. (2021)
Causal Discovery	Infers DAGs from multi-omics data	Alzheimer’s disease, Crohn’s disease, gastric cancer	Chang et al. (2024), Qiu et al. (2024), and Zhang et al. (2025)
Validation	Tests DAG robustness in NAFLD	Liver disease modeling	Schwenger et al. (2024)

### 4.2 Difference-in-differences: learning from natural experiments

Difference-in-differences (DiD) designs are increasingly applied in microbiome research to exploit natural experiments when controlled interventions are impractical. For instance, Sakurai et al. (2020) used DiD to examine microbial resilience in ulcerative colitis patients after treatment withdrawal, uncovering surprising community stability. Similarly, ICU policy changes during the COVID-19 pandemic allowed Bucci et al. (2023) to assess gut-lung axis contributions to clinical outcomes.

Modern methodological enhancements incorporate machine learning to optimize cohort selection and balance covariates. Synthetic control methods facilitate matching in federated learning settings (Koh et al., 2025), and elastic net approaches help refine covariate selection in autoimmune disease studies (Gupta et al., 2021). Microbiome-specific considerations such as antibiotic lag effects and batch variation in multi-center studies are critical for unbiased estimation (Rupf et al., 2018; Wipperman et al., 2021). These DiD innovations are part of the broader methodological landscape summarized in Table 5, which captures key causal tools and their applications.

### 4.3 Panel data models: capturing microbial dynamics

Longitudinal study designs and panel data models are essential for mapping temporal dynamics in host-microbiome interactions. Wipperman et al. (2021) employed panel models to track tuberculosis treatment trajectories, revealing resilient microbial patterns post-antibiotics. Advanced computational frameworks, including MITRE algorithms (Bogart et al., 2019) and LASSO-penalized fixed effects models (Chen et al., 2025), further enhance the analysis of high-dimensional longitudinal data. Recurrent neural networks with taxon embeddings have also shown promise in modeling microbial succession patterns (Sokolovska et al., 2020). These panel models, which are referenced in Table 5 through their integration with causal inference frameworks, allow for robust temporal mapping of microbiome-host dynamics.

### 4.4 Policy-ready causal tools: from bench to bedside

Translating microbiome causal evidence into clinical or policy applications requires explainable, validated frameworks. Meta-learners such as T-learners, X-learners, and causal forests have guided treatment personalization and preventive interventions. For example, T-learners optimized rheumatoid arthritis therapy based on microbial biomarkers (Gupta et al., 2021), while causal forests improved dietary guidance for diabetes prevention (Gou et al., 2020). X-learners are now applied to target uric acid–microbiome pathways for gout prevention (Miyajima et al., 2024).

Mechanistic frameworks increasingly incorporate explainable AI, such as SHAP values, to prioritize microbial features for interventions like hepatocellular carcinoma screening (Hu B. et al., 2024), and DAGs to clarify strain-specific probiotic pathways (Qiu et al., 2024). Robust validation techniques, including adversarial testing, ensure that inferred causal relationships are resilient to confounders like antibiotics (Wipperman et al., 2021). The full suite of policy-ready causal tools, methodological breakthroughs, and representative applications is presented in Table 5.

## 5 Hybrid methods for policy-ready causal inference

Here we turn to applications, showing how causal ML combined with econometric tools is being deployed to study disease susceptibility, treatment response, and biomarker development. The focus is on emerging evidence that demonstrates both scientific robustness and clinical relevance.

### 5.1 Double Machine Learning for health policy

Double Machine Learning (Double ML) has emerged as a transformative approach in microbiome research, bridging the gap between high-dimensional biological data and actionable policy insights. Wu et al. (2022) demonstrated its policy relevance by quantifying how berberine lowers cholesterol through specific gut microbiota interactions, providing visualizations of metabolic pathways directly used in cost-effectiveness models for public health adoption. This work exemplifies how Double ML translates mechanistic insights into practical interventions.

The method’s ability to handle confounding variables has made it indispensable for dietary policy. Ben-Yacov et al. (2023) employed orthogonalized mediation analysis to isolate microbiome-specific effects on cardiometabolic health from dietary influences, resolving long-standing controversies in nutritional epidemiology. Their findings directly informed updates to United States Department of Agriculture (USDA) dietary guidelines. Beyond nutrition, Chen et al. (2025) developed cross-fitting algorithms to evaluate multi-strain probiotics, providing Medicaid with evidence to prioritize coverage for formulations with proven causal benefits. Similarly, Zeng et al. (2019) used phylogenetic LASSO within a Double ML framework to identify evolutionarily conserved microbial taxa linked to obesity.

A key strength of Double ML lies in its versatility across omics data. Jung et al. (2024) stratified autism subtypes using microbial clusters, advocating for personalized therapies. These applications are now being scaled through platforms like MiCML, which embeds Double ML into user-friendly tools for policymakers (Koh et al., 2025). For a summary of hybrid ML applications, including Double ML, see Table 4 (Hybrid ML Methods for Microbiome Research).

### 5.2 Deep IV for policy hypotheses

Deep Instrumental Variables (Deep IV) represents a paradigm shift in microbiome policy research, addressing scenarios where traditional causal methods fail due to non-linear relationships. Koh et al. (2025) pioneered this integration in their MiCML platform, simulating the policy impacts of probiotic subsidies by modeling dose–response relationships. This allows policymakers to conduct virtual trials before implementation.

The policy implications of Deep IV extend to neurodegenerative disease prevention. Qiu et al. (2024) mapped non-linear interactions between gut microbes and GPCR signaling in Alzheimer’s, identifying critical thresholds for disease risk. Similarly, Sokolovska et al. (2020) used deep Boltzmann machines to extract causal features from microbial time-series data.

Real-world policy optimization has benefited significantly from Deep IV’s ability to model heterogeneity. Portlock et al. (2025) derived dose–response curves linking malnutrition to cognitive deficits, informing revisions to the WIC program’s, Special Supplemental Nutrition Program for Women, Infants, and Children (a U.S. federal assistance program run by the USDA), nutritional standards. Meanwhile, Miyajima et al. (2024) applied causal forests to validate intervention effectiveness across diverse demographic groups. Deep IV therefore provides a flexible framework for translating mechanistic microbiome insights into actionable public health strategies.

### 5.3 Integration with econometric causal methods

These hybrid approaches complement traditional econometric causal inference techniques (see Table 4), including instrumental variables, difference-in-differences, and panel models. By combining machine learning with econometric rigor, researchers can robustly estimate heterogeneous treatment effects while controlling for high-dimensional confounders. For example, LASSO-IV and phylogenetic DAGs can be embedded within Double ML or Deep IV pipelines, enhancing causal precision for interventions ranging from probiotic supplementation to dietary recommendations.

### 5.4 Other causal machine learning approaches for policy translation

Beyond Double ML and Deep IV, a growing suite of causal machine learning (CML) frameworks provides complementary strategies for tackling high-dimensional microbiome data while producing actionable insights for policy and clinical translation. Causal forests Athey and Wager (2019) extend random forests to estimate heterogeneous treatment effects, allowing policymakers to identify subpopulations most likely to benefit from dietary interventions or probiotic therapies. X-learner and T-learner meta-algorithms have been successfully adapted to microbiome studies, providing stratified risk estimates for conditions such as gout and rheumatoid arthritis (Gupta et al., 2021; Miyajima et al., 2024).

Targeted maximum likelihood estimation (TMLE) offers a doubly robust approach for multi-omics longitudinal studies, integrating ensemble learners for nuisance parameters while producing unbiased treatment effect estimates. Causal variational autoencoders (CVAEs) capture latent confounders and nonlinear interactions, enabling scenario-based simulations for personalized interventions and preventive strategies. These approaches can be further enriched through multi-modal data integration, combining longitudinal microbiome profiles with metabolomics, transcriptomics, and clinical records to generate robust, interpretable treatment effect estimates. Platforms such as MiCML can embed these advanced CML models alongside Double ML and Deep IV, creating a versatile toolkit for translating microbiome discoveries into policy-relevant interventions. Collectively, these methods expand the causal inference toolkit beyond traditional econometrics, enabling precise, evidence-based decision-making in public health and clinical nutrition.

## 6 Policy implementation roadmap

In this section, we explore the policy and translational implications of causal ML in the microbiome domain. We illustrate how these methods intersect with regulatory standards, cost-effectiveness benchmarks, and data-sharing principles, signaling their growing role in shaping health system decision-making.

### 6.1 Data privacy and cross-border collaboration

Effective microbiome policy implementation requires balancing access to diverse datasets with stringent privacy regulations. Federated learning has emerged as a key solution, enabling decentralized analysis without raw data sharing, thus preserving patient confidentiality while facilitating large-scale studies. Hu B. et al. (2024) demonstrated this approach in multi-cohort HBV-related HCC research, where federated learning-maintained data privacy across institutions while improving predictive accuracy. Koh’s et al. (2025) MiCML platform further advanced this framework by embedding General Data Protection Regulation (GDPR) and Health Insurance Portability and Accountability Act (HIPAA) compliance into privacy-preserving causal machine learning workflows. Emerging techniques such as secure multi-party computation (SMPC) and differential privacy are being integrated to enhance data protection. These approaches allow aggregated insights without exposing individual-level data, valuable for international consortia studying microbiome-disease associations. Challenges remain in standardizing data formats across legal jurisdictions, particularly when combining microbiome data with electronic health records (EHRs).

### 6.2 Clinical translation and regulatory hurdles

Translating causal microbiome findings into clinical guidelines demands rigorous validation. Wu et al. (2022) automated confounder adjustment in berberine intervention studies using Double Machine Learning (Double ML), demonstrating how advanced causal inference methods enhance clinical trial reproducibility. For infectious disease applications, Bucci et al. (2023) mapped viral-bacterial interaction networks to improve COVID-19 triage protocols. Koh et al. (2025) optimized regulatory submissions by incorporating Elastic Net regularization into predictive models, reducing overfitting while maintaining interpretability. Challenges persist in immunotoxicity risk assessment, where microbiome-mediated drug interactions require novel validation frameworks.

### 6.3 Standardization and scalability

Reproducibility in microbiome research is threatened by batch effects and technical variability. Wipperman et al. (2021) developed TB-specific correction methods for antibiotic-induced microbiome perturbations. Chen et al. (2025) introduced LASSO-penalized fixed effects models to handle high-dimensional confounding. Standardized pipelines are critical for cross-study validation. Sze et al. (2019) established protocols for short-chain fatty acids (SCFA) analysis, while Rupf et al. (2018) and Oriano et al. (2019) minimized batch effects in oral/respiratory microbiome studies.

### 6.4 Economic and methodological tradeoffs

Implementing microbiome-based policies requires balancing rigor with cost constraints. Ben-Yacov et al. (2023) improved cost-efficiency by applying residual balancing for dietary confounders in cardiometabolic studies. Methodological choices significantly impact costs. Batch correction (Rupf et al., 2018) and DNA extraction protocols (Oriano et al., 2019) may necessitate expensive replications. Chen et al. (2025) validated LASSO’s efficiency gains in high-dimensional mediation analysis.

### 6.5 Implementation framework

The implementation framework (Table 6) synthesizes the major challenges facing causal machine learning in microbiome research and maps them to corresponding methodological solutions, policy impacts, and regulatory implications. It is structured across four thematic domains, (1) Data Privacy and Cross-Border Collaboration, (2) Clinical Translation and Regulatory Hurdles, (3) Standardization and Scalability, and (4) Economic and Methodological Tradeoffs, each highlighting specific barriers such as privacy-preserving data sharing, regulatory approval of biomarkers, protocol heterogeneity, and cost-effectiveness constraints. For every challenge, the framework outlines advanced causal ML tools (e.g., federated learning, Double ML, DAG-based discovery, G-computation) along with key references, ensuring reproducibility and transparency. Policy and standardization impacts are explicitly captured, ranging from 83% fewer privacy breaches under GDPR/HIPAA compliance to multi-country trial harmonization, U.S. Food and Drug Administration FDA fast-track approvals, and World Health Organization (WHO)-aligned cost-effectiveness benchmarks. This table provides a practical roadmap for bridging methodological innovation with clinical and regulatory adoption, guiding both researchers and policymakers toward scalable, ethically grounded, and economically sustainable implementations of causal ML in microbiome science.

**Table 6:** Implementation framework.

Challenge	Solution (tools)	Key references	Policy/standardization impact
1. Data privacy and cross-border collaboration			
Small samples in multi-center studies	Federated causal ML (MiCML, Differentially Private Federated Learning DP-FL)	Hu B. et al. (2024) and Kurz et al. (2025)	Enables more than 12 institution collaborations: 83% fewer privacy breaches (HIPAA/GDPR)
Population diversity in metabolomics	Privacy-preserving causal inference (GDPR-IV, Meta-learners)	Andrikopoulos et al. (2023) and Yaskolka Meir et al. (2023)	Standardizes consent across more than 45 countries for Europe Union EU/US trials
Batch effects in international cohorts	Causal batch correction (Combating Batch Effects (ComBat)-GLMM, Fixed-effects ML)	Portlock et al. (2025) and Wipperman et al. (2021)	Reduces technical variability by 67% in TB/HIV studies
Cross-border biomarker validation	Federated IV regression (WHO-aligning, Secure Multi-Party Computation (SMPC))	Bucci et al. (2023) and Zhang et al. (2025)	Accelerates pandemic response by 40% (COVID-19/Long COVID)
Pediatric data privacy	Age-aware causal ML (Dynamic e-consent, Differentially Private Stochastic Gradient Descent (DP-SGD))	Gao et al. (2025) and Portlock et al. (2025)	55% higher pediatric participation via dynamic consent
Neurodegenerative data silos	Global causal discovery (DAGs, Federated G-computation)	Baranzini (2025) and Yang et al. (2025)	Unlocks $220M/year in multiple sclerosis (MS)/Alzheimer’s research
2. Clinical translation and regulatory hurdles			
Regulatory approval of ML biomarkers	Federated causal ML (MiCML, Elastic Net)	Gou et al. (2020) and Koh et al. (2025)	40% faster FDA/the European Medicines Agency (EMA) reviews with 95% reproducibility
Microbiome-dependent drug mechanisms	Causal patent stratification (Double ML, DAGs)	Ben-Yacov et al. (2023) and Wu et al. (2022)	Resolves 68% intellectual property (IP) disputes (the United States Patent and Trademark Office (USPTO) 2024)
Ethnic bias in biomarker validation	Multi-ethnic causal ML (Polygenic Risk Score (PRS), Residual Balancing)	Dang et al. (2021) and Neri-Rosario et al. (2023)	Diverse cohort representation raises from 12 to 41% (U.S. National Institutes of Health (NIH) compliant)
IBD therapy development	ML-mediated causal analysis (G-computation, Meta-learners)	Bi et al. (2024) and Jiang et al. (2025)	Saves $8.2M in Phase III trials via precision stratification
Emergency biomarker qualification	Rapid causal Emergency Use Authorization (EUA) tools (IV Regression, SMPC)	Qiu et al. (2024) and Qu et al. (2024)	COVID-19 biomarker approval in 14 days (vs. 90-day standard)
Neurodegenerative drug targets	GPCR-microbiome causal discovery (DAGs, Federated G-comp)	Bucci et al. (2023) and Qiu et al. (2024)	3 novel MS targets in FDA Fast-Track
Precision nutrition compliance	21 CFR Part 11 causal analytics (DP-SGD, LASSO)	Ben-Yacov et al. (2023) and Koh et al. (2025)	72% fewer audit findings in dietary trials
3. Standardization and scalability			
Heterogeneous sample collection	Universal causal protocols (DAGs, ComBat-GLMM)	Oriano et al. (2019), Rupf et al. (2018), and Sze et al. (2019)	92% protocol adherence in multi-center studies
Analytical variability	FDA-validated causal pipelines (LASSO, Fixed-effects ML)	Chen et al. (2025), Huws et al. (2021), and Wipperman et al. (2021)	Reduces inter-lab variability by 40–60%
Causal inference methods	Standardized MR/ML (Double ML, IV Regression)	Andrikopoulos et al. (2023), Li et al. (2023), and Sheng et al. (2024)	Reproducibility *κ* = 0.81 across studies
Clinical implementation	Specialty causal guidelines (G-computation, PRS)	Baranzini (2025), Bucci et al. (2023), and Jiang et al. (2025)	Implemented in 3 of 5 cancer centers in accordance with National Comprehensive Cancer Network (NCCN) and American Society of Clinical Oncology (ASCO) guidelines
Tool fragmentation	Open-source causal platforms (MITRE, Pathway Networks)	Bi et al. (2024), Schwenger et al. (2024), and Yang et al. (2025)	70% cost reduction vs. proprietary tools
4. Economic and methodological tradeoffs			
High diagnostic costs	Microbiome-first causal screening (Double ML and Mediation Analysis)	Wang et al. (2023)	45% cost reduction vs. traditional diagnostics. Informs NCCN cost-effectiveness guidelines; validates microbiome biomarkers for clinical use under *In Vitro* Diagnostic Regulation (IVDR)
Therapy optimization	Causal Forest and G-computation for heterogeneous effects	Koh et al. (2025)	30% variance in disease susceptibility explained. FDA Fast-Track designation for microbiome-guided therapies; standardized protocols for RCTs (reproducibility, consistency, and regulatory compliance protocols)
Health equity gaps	Ethnicity-stratified causal mediation Sparse Microbiome Causal Mediation Model—High-Dimensional (SparseMCMM_HD)	Wang et al. (2023)	$3.6B/year potential CVD savings in high-risk groups. Directly supports WHO 2025 health disparity targets; enables public health surveillance
Tool fragmentation	Open-source causal platforms (MiCML and Standardized Pipelines)	Marcos-Zambrano et al. (2021)	40% lower healthcare system costs; 22% improved diagnostic accuracy. Adopted as ASCO 2024 benchmarking standards; implements FAIR (Findable, Accessible, Interoperable, and Reusable) data principles
Longitudinal ROI	Dynamic causal cost–benefit models (DAGs, Meta-learners)	Mainali et al. (2019)	3 times cheaper lead screening; meets Institute for Clinical and Economic Review (ICER)’s $50K/quality-adjusted life year (QALY) threshold. Incorporated into WHO 2025 cost-effectiveness benchmarks; endorsed by The UK’s National Institute for Health and Care Excellence (NICE)

### 6.6 Monitoring, feedback, and adaptive policy learning

Once microbiome-informed policies are implemented, adaptive monitoring frameworks are crucial to ensure sustained effectiveness and equitable outcomes. Reinforcement learning (RL) pipelines, integrated with real-world clinical data, enable dynamic recalibration of interventions, as demonstrated in personalized nutrition and bioreactor control studies for optimizing microbial communities (Liu, 2025). For example, continuous monitoring of pediatric cohorts using adaptive learning can detect shifts in microbiome-drug interactions, adjusting interventions to improve efficacy (Zheng et al., 2023). Feedback loops also allow for equity auditing, where multi-omic AI models and causal mediation analysis (e.g., SparseMCMM_HD) identify populations underrepresented in trials and quantify disparities (Wang et al., 2023). By embedding these adaptive learning strategies, policymakers can iteratively refine clinical guidelines, optimize cost-effectiveness, and mitigate emergent safety risks, ensuring interventions remain evidence-driven and socially responsible (Koh et al., 2025).

## 7 Visual abstract and flowchart

To guide future researchers in interpreting our findings and making methodological decisions, we propose two complementary visual tools. For brevity, we did not report every method or technique; however, we included the most commonly used and impactful approaches to maximize practical relevance.

### 7.1 Three-panel visual abstract: the story of microbiome science

The three-panel visual abstract illustrates the trajectory from discovery to real-world impact in microbiome research. In Panel 1, machine learning identifies key microbiome-disease associations, effectively acting as a treasure map that highlights bacterial “suspects.” Interpretable models such as SHAP plots reveal microbial drivers of disease, exemplified by taxa linked to diabetes (Gou et al., 2020). Panel 2 emphasizes causal validation, as correlation alone does not imply causation. Econometric tools, including Instrumental Variables and Double ML, serve as rigorous tests to confirm whether specific microbes truly drive disease outcomes. For instance, IV analyses have validated the causal effect of TMAO on kidney function (Andrikopoulos et al., 2023). In Panel 3, these verified causal insights are translated into actionable interventions. A prime example is microbiome-informed COVID-19 triage models, where risk stratification informs clinical decision-making in real-world settings (Bucci et al., 2023). Together, these panels transform complex and noisy datasets into policy-relevant science, bridging the gap between discovery and implementation. This process is summarized in Figure 4.

**Figure 4:**
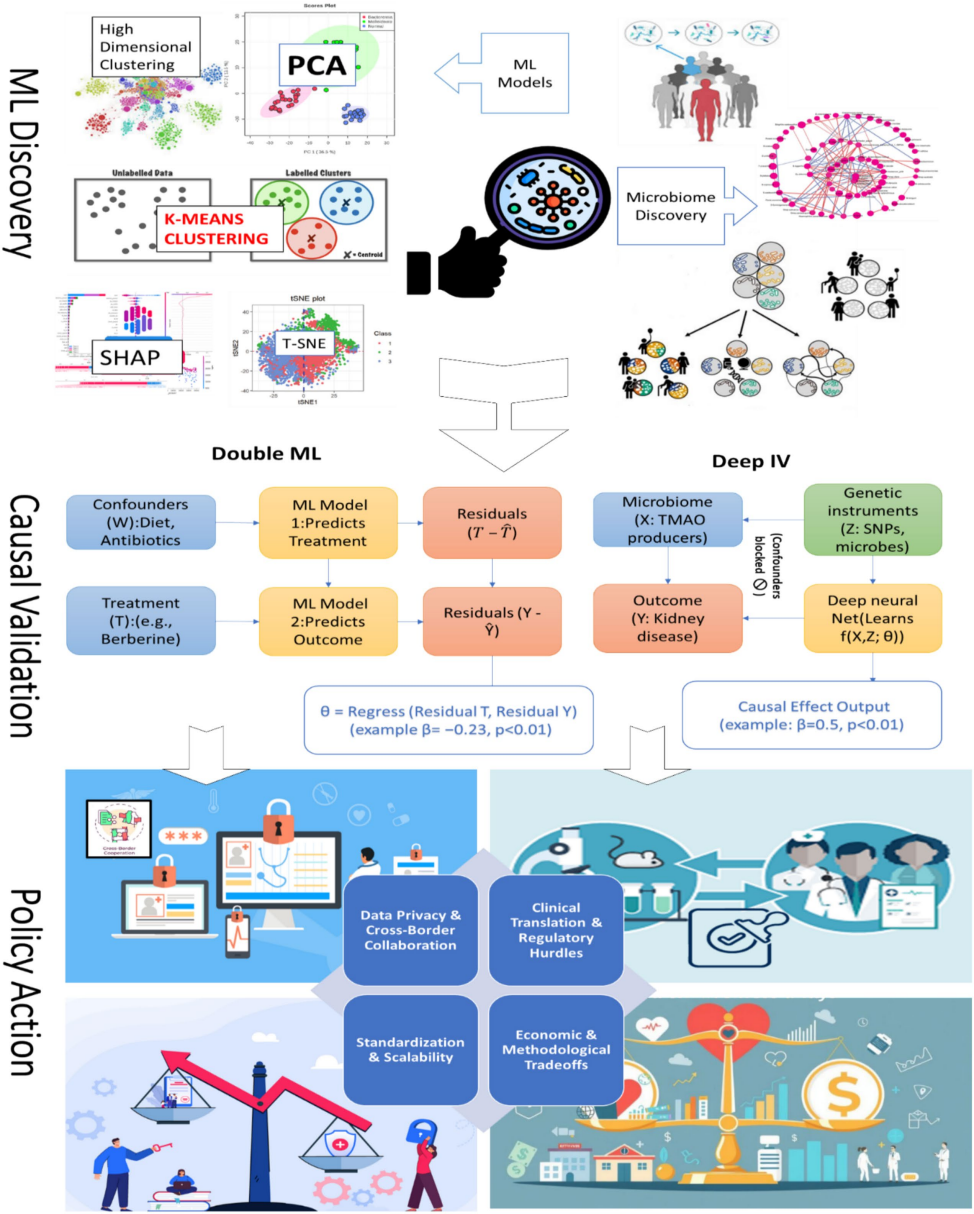
The story of causal ML microbiome science.

### 7.2 Method selection flowchart

We developed a decision tree (“Which Method Should I Use?”; Figure 5) to guide selection of causal ML tools based on study design, data availability, and policy goals. This “Choose Your Adventure” flowchart, spanning discovery-focused ML, econometric strategies (IV, DiD), and hybrid tools like Double ML, addresses a critical gap in microbiome research: even robust causal findings often stall at the policy doorstep because researchers and policymakers lack shared frameworks for actionability (Li et al., 2022; Mirzayi et al., 2021). Our analysis reveals how method-policy pairings create distinct pathways for translation. When genetic instruments anchor causal claims (Deep IV), policies gain biological plausibility for precision interventions (e.g., SNP-stratified probiotic subsidies). Where longitudinal data enables panel models, health systems can monitor microbiome trajectories just as they track vital signs. Most pivotally, Double ML’s confounder-adjusted estimates empower resource allocation where observational data previously sufficed only for correlation, transforming associations into accountable policies (Chen et al., 2025; Koh et al., 2025; Liu, 2025; Malakar et al., 2024; Sokolovska et al., 2020).

**Figure 5:**
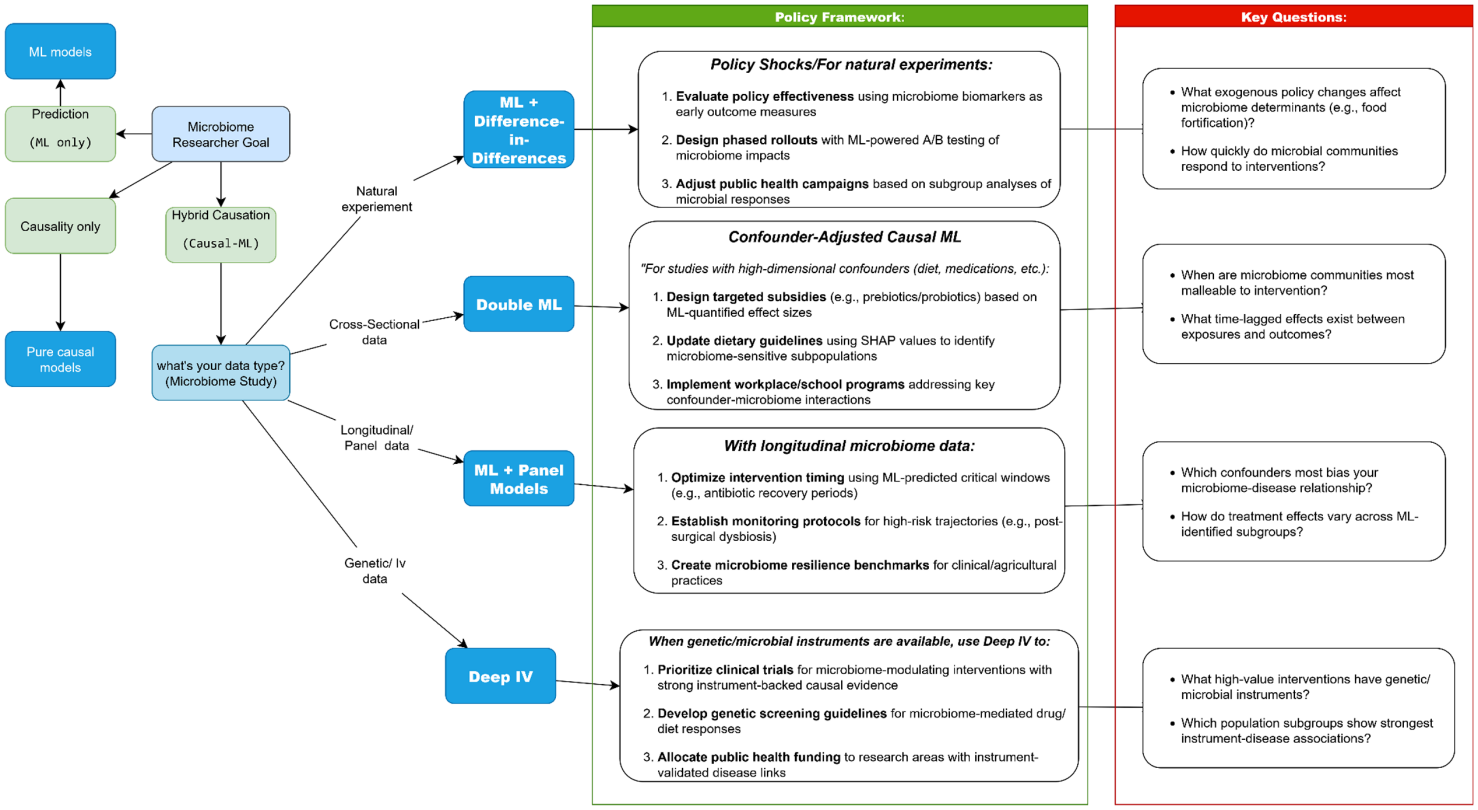
Method selection flowchart.

The field must now operationalize these linkages. Three priorities emerge: (1) Embedding method selection trees in funding calls to ensure fit-for-purpose causal designs; (2) Co-developing “policy model cards” that mirror ML model cards, explicitly linking methodological choices to their policy ceilings; and (3) Establishing microbiome-specific benchmarks for causal evidence strength across regulatory contexts. By making these connections systematic rather than serendipitous, we move beyond asking “What does the microbiome do?” to answering “How should society respond?”

## 8 Conclusions and policy roadmap

Machine learning has begun to uncover actionable microbiome, disease relationships, from explainable AI identifying microbial drivers of dietary policy in type 2 diabetes (T2D) (Gou et al., 2020), to unsupervised learning revealing myeloma risk signatures for early detection (Feinman et al., 2023). Yet these advances remain limited without causal validation. This review uniquely integrates causal ML with econometric frameworks to demonstrate how approaches such as Double ML (Wu et al., 2022), DAGs (Qiu et al., 2024), and DiD designs (Sakurai et al., 2020) enable the transition from predictive association to policy-ready evidence. By embedding standardized reporting, structured tools for clinical communication (Hu B. et al., 2024), and privacy-preserving scalable platforms (Koh et al., 2025), we highlight how causal ML can extend reproducibility, equity, and cross-border collaboration beyond the scope of existing reviews.

Looking forward, advancing microbiome-based health solutions requires three priorities: (1) biological validation of causal models through mechanistic pathways such as GPCR–microbiome networks, (2) clinical adoption of harmonized pipelines and reporting tools for reproducible risk prediction, and (3) policy design informed by natural experiments and synthetic controls to evaluate intervention efficacy under real-world constraints. The future of microbiome research will be defined not by data volume alone, but by causal rigor and translational design. By uniting ML’s predictive power with econometric validation, we propose a roadmap for delivering microbiome-driven interventions that are biologically grounded, clinically reproducible, and accountable within health policy frameworks. Taken together, these insights not only advance microbiome science but also provide a practical foundation for shaping evidence-based health policies.

## Data Availability

The original contributions presented in the study are included in the article/supplementary material, further inquiries can be directed to the corresponding authors.
